# From Anomaly Detection to Defect Classification

**DOI:** 10.3390/s24020429

**Published:** 2024-01-10

**Authors:** Jaromír Klarák, Robert Andok, Peter Malík, Ivan Kuric, Mário Ritomský, Ivana Klačková, Hung-Yin Tsai

**Affiliations:** 1 Institute of Informatics, Slovak Academy of Sciences, 845 07 Bratislava, Slovakia; robert.andok@savba.sk (R.A.); p.malik@savba.sk (P.M.); upsyrima@savba.sk (M.R.); 2 Department of Automation and Production Systems, Faculty of Mechanical Engineering, University of Zilina, 010 26 Zilina, Slovakia; ivan.kuric@fstroj.uniza.sk (I.K.); ivana.klackova@fstroj.uniza.sk (I.K.); 3 Department of Power Mechanical Engineering, National Tsing Hua University, Hsinchu 30013, Taiwan; hytsai@pme.nthu.edu.tw

**Keywords:** automation, defect detection, anomaly detection, deep learning, autoencoder, clustering, visual inspection

## Abstract

This paper proposes a new approach to defect detection system design focused on exact damaged areas demonstrated through visual data containing gear wheel images. The main advantage of the system is the capability to detect a wide range of patterns of defects occurring in datasets. The methodology is built on three processes that combine different approaches from unsupervised and supervised methods. The first step is a search for anomalies, which is performed by defining the correct areas on the controlled object by using the autoencoder approach. As a result, the differences between the original and autoencoder-generated images are obtained. These are divided into clusters using the clustering method (DBSCAN). Based on the clusters, the regions of interest are subsequently defined and classified using the pre-trained Xception network classifier. The main result is a system capable of focusing on exact defect areas using the sequence of unsupervised learning (autoencoder)–unsupervised learning (clustering)–supervised learning (classification) methods (U2S-CNN). The outcome with tested samples was 177 detected regions and 205 occurring damaged areas. There were 108 regions detected correctly, and 69 regions were labeled incorrectly. This paper describes a proof of concept for defect detection by highlighting exact defect areas. It can be thus an alternative to using detectors such as YOLO methods, reconstructors, autoencoders, transformers, etc.

## 1. Introduction

Defect detection in visual data can be considered a separate part of object detection used primarily as part of inspection systems in industrial applications. The applicability of inspection systems is in the field of Industry 4.0 and the creation of cognitive control systems. Inspection processes may vary from inter-operational to final quality control of manufactured products [1,2]. The quality assessment or inspection objective can be diverse, from measurement and control procedures to defect monitoring. Many object detectors were proposed in the past. They can be divided into single-step or two-step detectors. YOLO [3,4,5] and SSD [6] are single-step detectors where the prediction of the final class and localization of the object are performed in a single step. The advantages are architectural simplicity, regularity with easy end-to-end training, and higher FPS; however, the cost is lower localization precision and recall. The two-step detectors use the additional step at the beginning in order to find regions of interest (RoIs) which are subsequently classified in the second step. This improves both the localization precision and the recall at the expense of increased computation and complexity. R-CNN [7], fast R-CNN [8], faster R-CNN [9], mask R-CNN [10], and cascade R-CNN [11] are examples of known two-step detectors. Mask R-CNN even includes the ability to classify the objects on the pixel level (segmentation) and is proven to improve the accuracy in end-to-end learning together with classification and localization. Cascade R-CNN repeats the classification step several times, with increasing localization requirements, improving the accuracy further [3,7,9,10]. Visual pattern detection is one of the most researched problems with varied accuracy and many applications. There are many goals that are mostly competing. The main goal is to maximize the ability to correctly detect objects of interest in visual data. The second goal is to perform the prediction efficiently. For industry applications, there are minimal frame rate requirements of 30, 60, or more frames per second (FPS), depending on the specificity of the task. The maximal latency of the prediction is another crucial parameter of industrial applications required for many real-time visual detection tasks. More efficient detectors save energy and can be run on less expensive computation resources. Anomaly detection offers visual detection with a focus on differences without defining a closer category or type of detected anomaly. This approach is used in many cases where anomalies are filtered through autoencoders, U-nets, or similar methods. It prevails in the field of medical applications, especially in machine evaluation of MRI and CT images [12,13,14].

In our research, we have focused on the detection of defects, including various shapes contained (or not) in the training dataset, that may differ in orientation, position, color, display, etc. This is because the defect is a random element with not exactly defined structures. Therefore, standard models based solely on the detection of trained patterns may be insufficient in the practical application of inspection systems. The second generally used approach mentioned in the literature is based on the comparative method, using an autoencoder, a U-network, or a visual transformer, with the purpose of having a trained network capable of reconstructing the input image according to the trained pattern, thereby suppressing possible anomalies in the test image. When comparing the generated image and the tested image, anomalies become visible. This is the essence of the first part of the presented defect detection system. From the point of view of applicability, this method does not give information about the kind of anomalies manifested as differences between the tested and reconstructed images. In order to determine the type of anomaly, additional steps should be completed. Selecting a suitable clustering algorithm is crucial for the correct separation of the differences according to their position into related categories so that it is possible to determine which pixels correspond to which anomaly. Since it is not possible to estimate how many anomalies will be recorded from the autoencoder, it is necessary to choose a clustering algorithm separating the data not according to a predefined number of classes but, rather, according to the data structure. From this point of view, the most suitable algorithms are DBSCAN and OPTICS. DBSCAN has been chosen due to its ability to separate the differences into relevant clusters. Based on the clusters, the areas of interest named as the RoIs were defined, which enter the third phase of the system, the so-called classifier for determining the type of anomaly. The pre-trained Xception network was used as a classifier.

The question is whether it is possible and efficient to focus on exact areas of defects, building an alternative to the clear supervised methods (for instance, YOLO approaches) or to the reconstructor models only (such as autoencoders, U-nets, or transformers) in defect detection and whether only the supervised methods are sufficient enough for defect detection, as well as what are the fundamental differences in defect detection from object detection.

The main contributions and support hypotheses in the proposed U2S-CNN are as follows:

Unsupervised method—anomaly detection: design a custom autoencoder to reconstruct an input testing image to its more idealized version without anomalies and use its output to detect and locate anomalies in the test image.

Unsupervised method—clustering: separation of the differences in anomalies and regions of interest by using the DBSCAN clustering method, independent from the defined number of clusters and their shapes or patterns. The issue being solved is the following: how many anomalies or defects occur in the tested image?

Supervised method—classification: classification of anomalies in the way of regions of interest to pre-trained labels. The issue being solved is the following: what type of anomalies occur in the tested image in the way of the detected anomaly?

## 2. Related Works

The defect detection was gradually implemented simultaneously with the development of general methods for detecting objects or patterns. One of the first applications of the R-CNN method for defect detection is described in [15]. The detection method using R-CNN is based on the definition of 2000 region proposals that are classified. Defining these 2000 regions depends on the algorithm used to extract the given regions. In the case of a homogeneous image or the extraction of a specific image, it can be problematic to correctly capture or delineate a specific pattern.

The detection of defects using the fast R-CNN method is described in [16], where four types of defects were detected on discs of passenger car wheels. The achieved precision was from 68.5% (oil pollution) to 74.9% (scratch). In the case of error detection, the faster R-CNN method was used more often. For example, an improved faster R-CNN method is described in [17]. The authors use a specific type of data from the ground-penetrating radar device, which monitors the condition of the rails. They used a Caffe-based faster R-CNN to detect errors in this specific data. The result is the detection of four types of defects, where the detection accuracy expressed by the F-Score was from 75.8% to 83.6%, and the precision was 85.2%. Another faster R-CNN-based method for detecting defects in fiber paper tubes is presented in [18]. The data acquisition hardware consisted of a line camera and a red line illumination. Defect detection was performed by the faster R-CNN + ZF method. The precision was from 47.0% (internal joint detection, 15 pics) to 97.8% (external joint detection, 75 pics). The detection of tire bubble defects on digital shearography data is described in [19]. Bubble detection was divided into detection on the sidewalls of the tire and on the tread of the tire. Detection accuracies of 86.87% (tire sidewall) and 89.16% (tire tread) was achieved. The paper with a description of detection and classification applied for metallic surface defect detection is in [20]. In this work, an autoencoder with connected layers is applied to define different areas. The differences are cropped and classified into the three types of defects. A specific approach for defect detection was chosen in [21]. The work describes a two-phase approach, where the first phase is a segmentation network, and the second phase is a decision network. The segmentation network was evaluated on DeepLabv3+ and U-net architectures. This part of the designed system defines the defect area. The results were achieved with a precision from 96.1% to 99.9%. A similar work [22] demonstrated the results on the industrial databases DAGM, KolektorSDD, and Severstal Steel Defect. In a supervised manner, an accuracy from 91.88% (DAGM dataset) to 100.00% (KolektorSDD dataset) was achieved. Another specific approach is based on a residual convolutional neural network. The application is for detecting defects occurring on printed circuit boards (PCBs) [23]. Another category of error detection is an approach based on autoencoders capable of generating a sample image, e.g., [24]. The resulting applications are for detecting defects such as scratches, surface cracks, and caverns. Using U-net with the Xception architectural modules achieved a precision of 0.87. Another work (Kim et al., 2021) is based on an autoencoder with custom architecture [25]. A more advanced anomaly detection principle can also be based on visual transformers, which is presented in [26], where anomaly detection reached between 78.9 and 100.0%, depending on the category.

The solution with the improved YOLOv4 model reached a mean average precision of 94.5%, supporting the feasibility of the YOLOv4 model for this type of task. A frame rate of 42 FPS was applied using an NVIDIA GeForce GTX 1080Ti [27]. The solution was based on a transformer and performed on MVTec and their dataset, where the performance was from 99.42 to 99.92% [28]. The application of the U-Network architecture performer on the MVTec dataset is described in [29]. The AUC score reached 98.80% at the image level. The principle of work is based on the defined differences between the input and generated images. A summary of the results is presented in Table 1.

To sum up, in scientific contributions, the chosen approach of searching for defects is based on detection principles such as R-CNN methods or possibly based on segmentation methods. The second branch is the detection of anomalies in the data obtained by comparing the input with generated or filtered images. In this case, primary U-networks, autoencoders, and visual transformers are used. This approach is mainly applied in the field of industry [25] or in the field of medicine, especially the detection of anomalies in MRI images [30,31].

The main topics about finding defects in inspected objects for industrial applications are in the two dominant approaches: supervised methods—detectors for defect detection or unsupervised methods—reconstructors using unlabeled data in the way of autoencoders or U-nets. The detectors achieve good results, but in our opinion, the usage of detectors is not enough to detect a large number of defect types, especially the defects not contained in a dataset. The unsupervised methods do not provide enough information about the specific area of defects or the label of these defects. We see limits of these methods dealing with an idea of unknown defects or unlabeled defects in using reconstructors. We see a solution in the combination of the advantages of both approaches into one system.

Our effort is to summarize a comprehensive approach to solving the problem of detecting defects in data and solving the primary shortcomings of both solutions, where the area of anomaly detection is supplemented by another part, namely the clustering of differences and the classification of specified areas. In the area of defect detection based only on detection systems of the faster R-CNN type and similar, there is an effort to focus on the detection of defects not contained in datasets and thus go beyond the issue of detecting defects of non-specific shapes, coloring, topology, and the like. A very interesting two-phase approach is presented in [21]. The premise of the problem is not to focus on limited defect datasets. This is due to the different display or structure of the defects. In the work [32], the emphasis is on the generality of the shape of defects that may not be included in the dataset. A better solution to general defect detection is based on the differential method, where generative or predictive methods such as autoencoders, visual transformers, or recurrent networks are used to filter out all differences. In this approach, the focus is not on detecting defects but on training the correct patterns. However, the output from such a system does not include sufficient information about the location and type of defect. Based on these issues, the detection system proposed is designed to combine the essence of both methods, while these methods are supplemented with the DBSCAN algorithm as a category of unsupervised learning to significantly increase the cognitive ability of the detection system.

## 3. U2S-CNN Three-Phase Defect Detection System

**Figure 1:**
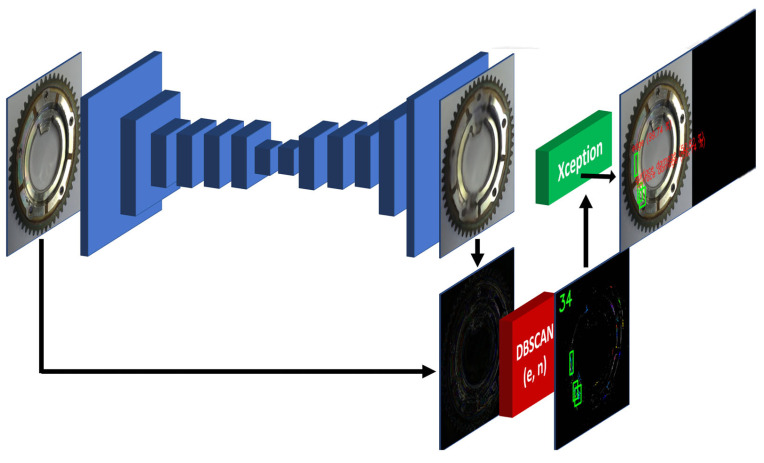
Illustration of method “from anomaly detection to defect classification”.

The defect detection system designed is a three-phase detection system (Figure 1), where the primary cognition of the system is based on the use of algorithms in the following manner:

First phase: Unsupervised learning–anomaly detection–defining the right areas and focusing on anomalies in the data.

Second phase: unsupervised learning–anomaly clustering–defining clusters from anomalies where the number, orientation, color, display, etc. may differ.

Third phase: supervised learning–anomaly classification–classification of areas of interest into predefined classes, either a correct part that was not properly reconstructed or an occurring defect.

The resulting detection system can be described as a U2S-CNN network developed for defect detection. The following sections will describe each phase in detail. The dataset used in this study was obtained in the laboratory by a 12 Mpx camera. The capture of objects was performed in general angles without additional light conditions. These images were cropped and resized to create the training and test data (224 × 224) for the first phase. For training the classification, the dataset of defects was made.

## 4. Anomaly Detection

**Figure 2:**
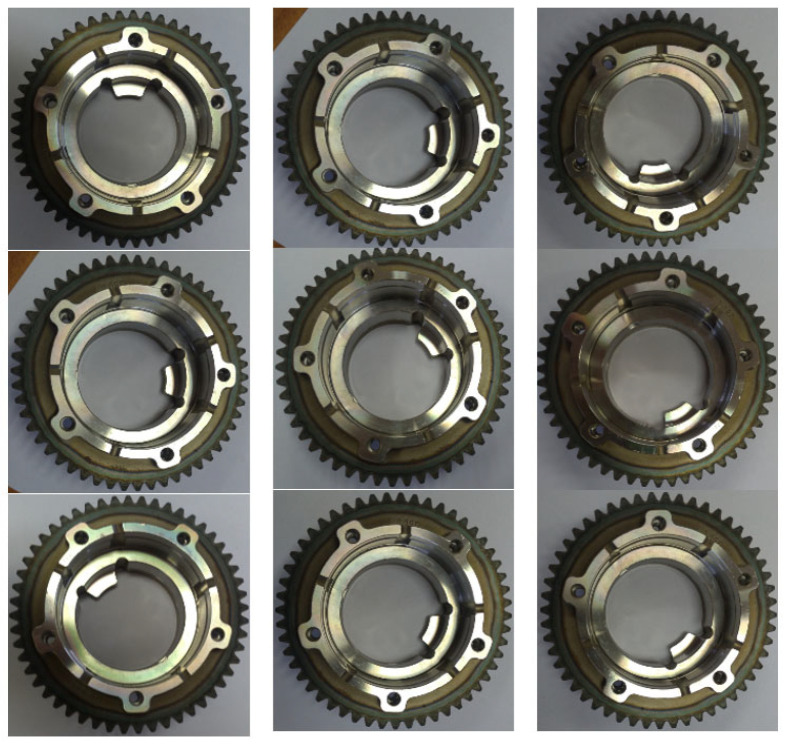
Examples of training dataset for the autoencoder.

Anomalies can be defined as differences in data that are created by comparing test data to expected data. Several methods, such as autoencoders, U-net visual transformers, and the like, can be used for this purpose. From the point of view of the application and focusing on anomalies, the primary goal is to detect these anomalies using the autoencoder. The Convolution, Maxpooling, and UpSampling layers of the autoencoder will learn to suppress the anomalies in the learning process. A trained autoencoder is able to reconstruct the test data by replacing correct or at least improved areas according to training patterns. The goal is to achieve the best possible reconstruction of the test image according to the trained patterns. The basic premise is to ensure enough precisely defined patterns for training (Figure 2). The base dataset consists of cropped images of a gear wheel of the same type due to the specialization of this application for industrial purposes. Autoencoders fall into the category of convolutional neural networks. The design of the convolutional neural network is built with Tensorflow [33], and primarily, the following layers are utilized:


BatchNormalization;Conv (2D);MaxPool (2D);UpSampling (2D).


**Figure 3:**
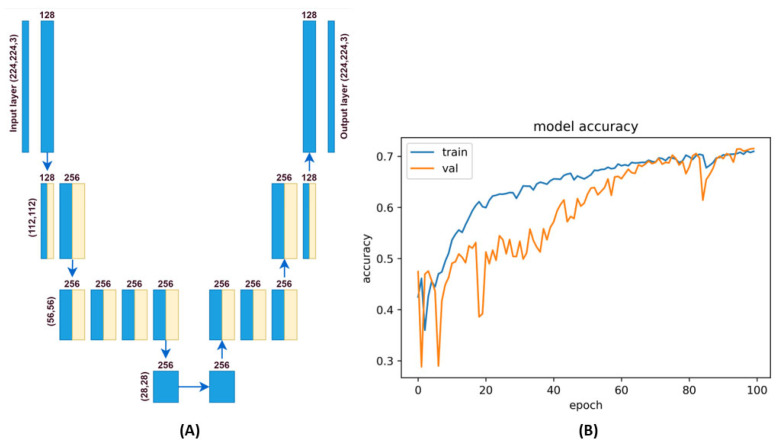
Architecture of the autoencoder and its training process. (**A**)—architecture of autoencoder network, (**B**)—training process of autoencoder.

The architecture of the autoencoder used is defined in Figure 3B. This type of network is an asymmetric autoencoder, where filters are trained in the latent space due to the suppression of possible defective areas during the image reconstruction process. So, the number of filters was set to 256 in the latent space. The neural network training parameters are listed in Table 2, based on which it is also possible to assume the difficulty of the training or predictive process. The ‘adam’ optimizer and “mean_squared_error” loss function were used for training. The training was performed on 78 images with correct samples in 100 epochs. The result of the training process is shown in Figure 3B. The training data and output data (y) were used the same. So, the autoencoder trains to patterns of correct samples. The training accuracy was achieved at 72.02%. Such a network is sufficient to generate a sample image in the conditions of this research.

## 5. Clustering to Regions of Interest

**Figure 4:**
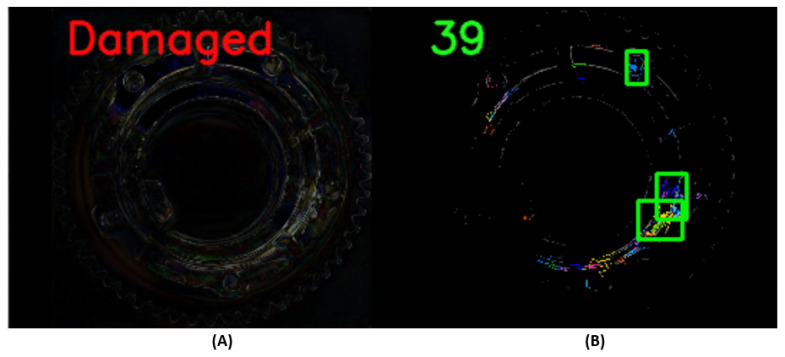
The detected anomalies clustered to regions. (**A**)—image of found differences, (**B**)—clustered differences to clusters and made regions of interest (ROI).

In the previous phase, anomalies defined as differences in test and reconstructed test data were detected, see Figure 3. The majority of the published works about anomaly detection usually end at this stage. Furthermore, these works do not specify what to do with this information. In this work, the focus is on anomaly detection and also on the identification of detected anomalies. The main point of this phase is the distribution of detected differences according to individual labels corresponding to anomalies on the test object. It is impossible to say how many anomalies, what shape, location, display, and topology may occur on the test object. Clustering algorithms as part of Unsupervised learning are suitable to solve this issue with an unknown number of possible occurring anomalies. Most clustering algorithms are based on methods of separating points in 2D or 3D space to a number of clusters defined before, for instance, the K-means method [34]. This way is not sufficient because this issue needs to separate many different numbers of points in every possible position to a separate non-predefined number of clusters. The simple description of the problem includes the unknown number, positions, size, and shape of these defects. For this reason, it is necessary to perform separation of the differences (in the way of different pixels) to cluster. For this reason, we decided to utilize existing clustering algorithms implemented in the scikit-learn library [35], which do not require the number of clusters to be predefined but work on the principle of the separation of pixels according to density relations. For this purpose, the suitable clustering methods are as follows: density-based spatial clustering of applications with noise (DBSCAN) or ordering points to identify the clustering structure (OPTICS) [36]. The DBSCAN algorithm was selected and used in this work. For this algorithm, two parameters have to be specified. The parameter epsilon (e or eps) is set as the radius of the searched space, and the parameter minimum number of samples (min_samples) is set as the minimum number of points in the searched space. Based on this, the points are divided into three categories: core points, boundary points, and other points or noisy points. In this work, parameters for the DBSCAN algorithm were defined: eps = 5 and min_samples = 5. A clustering result example is shown in Figure 4. The tested gear is shown, which was evaluated as damaged in the previous phase. The differences in RGB were transformed into monochrome expressions as the RGB mean. As a result, 39 clusters were recorded in this test pattern. A region of interest (RoI) can be defined from these clusters. Most of the clusters correspond to slightly blurred areas that were assimilated in the reconstruction process. For this reason, clusters containing at least 30 points were further used. In Figure 4, three such clusters are shown.

## 6. Classifying the Regions of Interest to Categories

**Figure 5:**
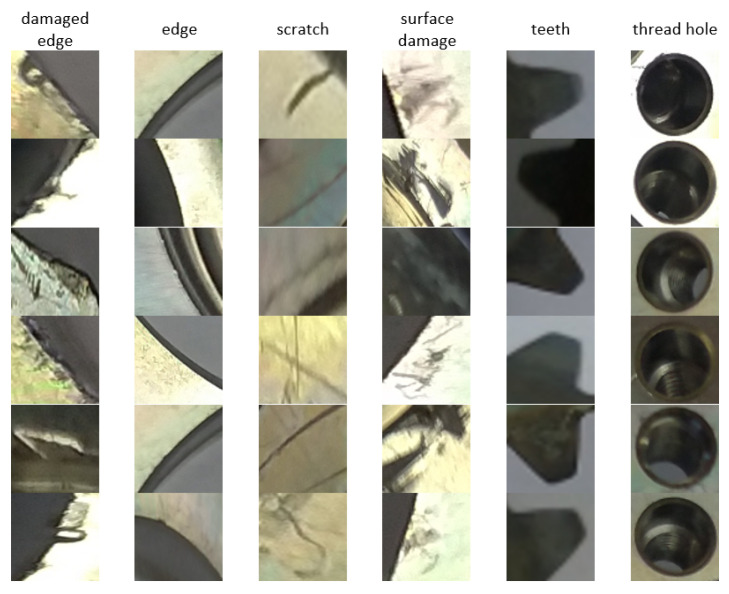
Examples of training dataset for the classification procedure.

In the previous phase, RoIs were defined using the clustering algorithm based on the differences from the autoencoder. This phase is performing the classification of RoIs into predefined classes. Based on the data, six basic types of objects were specified, where three types belong to defects, namely, damaged edge, scratch, and surface damage. The other three types belong to standard parts of a gear, namely edge, teeth, and thread hole. Such a division was chosen due to the possibility of incorrect reconstruction of the correct part of the inspected object. An example of the training dataset is shown in Figure 5. The number of samples for the training and validation dataset is shown in Table 3.

**Figure 6:**
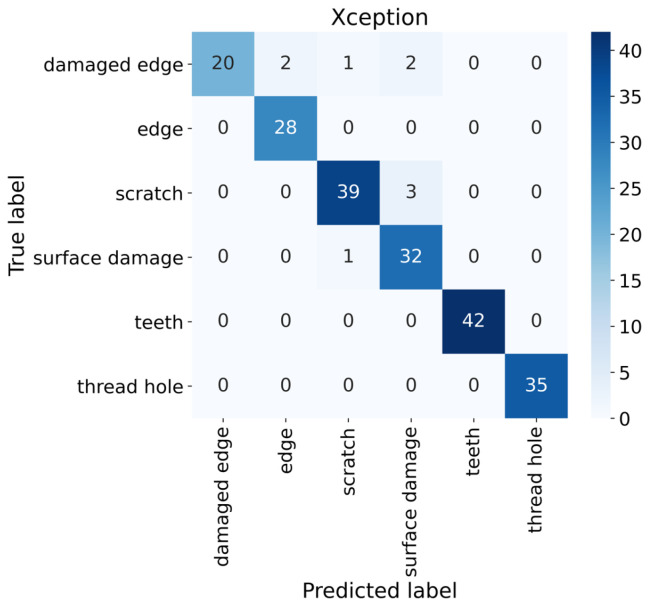
Confusion matrix of Xception model on validation dataset.

The third part of this work focuses on defect classification. In this phase, simple classification can be used since the anomalies and their localization are precisely defined from the previous phases. It is convenient to use the transfer learning method in the form of a pre-trained model for classification [37]. From the list of pre-trained models from the Tensorflow library [38], the Xception model was selected. This model is one of the best classification models, achieving very good results for different types of classification tasks [39,40]. The input dimension was set to 71 × 71 × 3, and the number of outputs to 6. The Xception neural network has 71 layers, and the parameters of the Xception are listed in Table 4. The SparseCategoricalCrossentropy for the loss function and the Adam optimizer were chosen. The number of epochs was chosen to be 100. The training accuracy reached 100%. Model validation was performed on 205 samples shown in Figure 6, where the validation accuracy reached 95.61%.

## 7. Results

**Figure 7:**
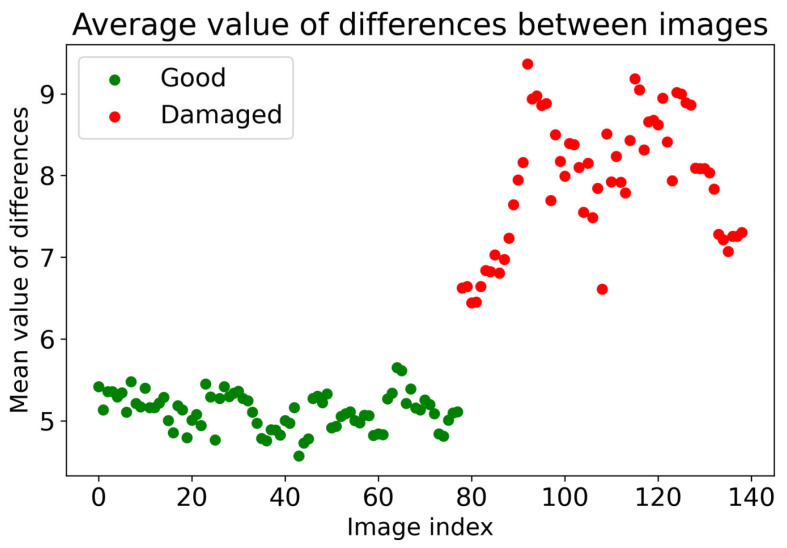
Validation of 1st phase of U2S-CNN.

Evaluation of the correctness of the inspected object is already possible in the 1st phase of the U2S-CNN network, where, based on the differences, it is possible to assume that there are anomalies on the inspected object. The validation of the first phase of the U2S-CNN network was performed on 144 samples shown in Figure 7, where the points represent the average value of the detected differences, while the green points below the experimentally set value of 5.5 represent the controlled samples without detected anomalies. The average values higher than 5.5 represent the high possibility of occurring anomalies in the inspected objects, most likely in the form of defects.

**Figure 8:**
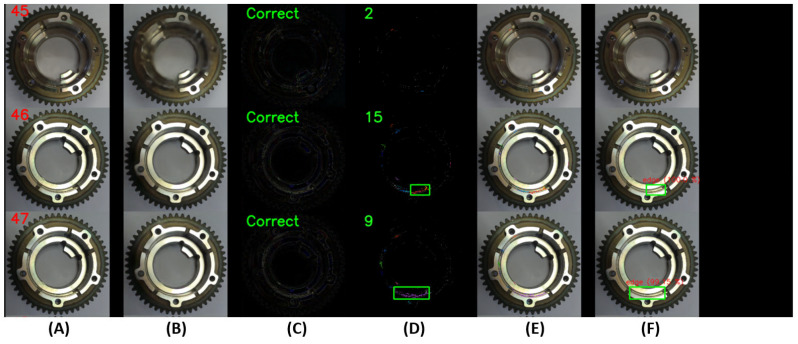
Illustration of working U2S-CNN network with correct samples, where (**A**)—input image, (**B**)—generated image by autoencoder, (**C**)—differences between input and generated images, (**D**)—clustered differences with shown regions of interest, (**E**)—merged input image and detected clusters, and (**F**)—labeled anomalies.

**Figure 9:**
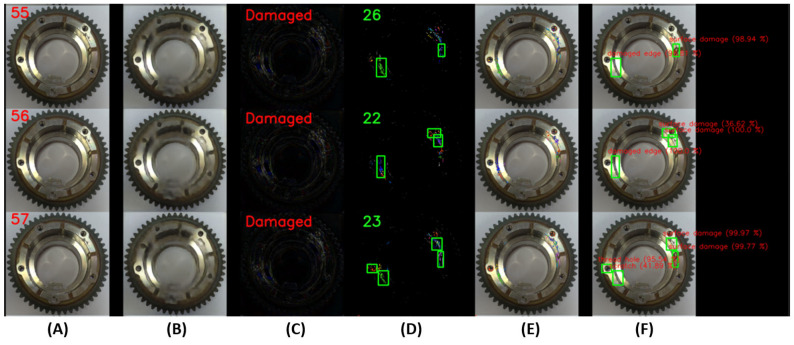
Illustration of working U2S-CNN network with damaged samples, where (**A**)—input image, (**B**)—generated image by autoencoder, (**C**)—differences between input and generated images, (**D**)—clustered differences with shown regions of interest, (**E**)—merged input image and detected clusters, and (**F**)—labeled anomalies.

An example of the operation of the U2S-CNN network is shown in Figure 8 and Figure 9, where the sequence of operation of the U2S-CNN network is shown. The input image (A) is processed by the autoencoder in the first phase (B). Differences are expressed based on the input and generated image (C). Based on these differences, it is possible to determine whether the image is correct or shows traces of anomalies or damage and requires the next inspection steps. Next, the differences enter the second phase, where they are separated into clusters using the DBSCAN algorithm (D). The number of detected differences is displayed as a green number. Clusters with more than 30 points are defined as anomalies and form RoIs for the third stage. Highlighting of the detected anomalies in the input image is shown in (E) (best seen in color and zoomed). Subsequently labeled RoIs are shown in (F).

The evaluation of the U2S-CNN network is described in Table 5. The evaluation is performed on two types of samples. The reconstruction reached an industry-acceptable result. All correct samples were defined as correct in the reconstruction process. Altogether, 423 clusters for 78 images were detected in the clustering process. From them, there were 16 regions of interest, which contained more than 30 pixels. In the classification process, there were seven regions labeled correctly, and nine regions were labeled incorrectly. In tested samples, there were 1920 clusters detected for 61 samples, and 177 RoIs were defined. In the classification process, 108 RoIs were classified correctly, and 69 RoIs were classified incorrectly. In total, 142 of 205 damaged areas were undetected. The main problem was to identify small defects correctly, e.g., small scratches or defects visually very close to the surrounding areas. More experimental work is necessary to find a suitable combination of light sources under specific angles that will increase the contrast and improve the detection capability. Light experimentation is out of the scope of this work and will be addressed in the future. This summarizes the complete results, including the manual review of the U2S-CNN network [41].

## 8. Discussion

In this work, a proof of concept for a hybrid system for defect detection built on unsupervised and supervised methods and combining their advantages was introduced. This study was performed on a dataset of 139 images for autoencoder consisting of 78 correct samples and 61 tested samples. For the next study, it is appropriate to consider a larger dataset prepared using artificial light for capturing photos in the environment of the black box and with a larger size of images. From such data, it will be possible to obtain higher quality and improved results of reconstruction, which is crucial for achieving good detection of defects. It is necessary to adapt light conditions to the shape and topology of the object being inspected, mainly to prevent the appearance of shadows. The other issue of application defect detection is connected with time dependency to perform this task. In this paper, this area is not explored. For further work, the monitoring of the time parameter and the use of optimized algorithms are planned to obtain faster performance of software and better usage of computational power. These tasks are planned for the further evolution of this solution, where an architecture and system of training process of models in supervised and unsupervised models will be adapted.

## 9. Conclusions

In this paper, a system designed for defect detection is presented. It is composed of three parts, where two parts are trained on an adequate dataset of data. In the first step, the cropped data in Figure 2 were used to train the autoencoder. In this way, it is possible to demonstrate the possibilities of anomaly detection when applying such a system very well for industrial purposes in limited conditions. This term means a precisely defined object with adequate sensing conditions, for example, lighting and the position of the sensing device in relation to the object, etc. It is especially good to highlight the cognitive nature of the proposed system, not being limited to the detection of defined patterns but to the detection of anomalies that are classified into individual classes. The classification of differences into anomalies is ensured by a clustering algorithm, which significantly increases the cognitive ability of such a system. In its current form, this system is based on the use of an autoencoder. For other specific purposes, the autoencoder can be replaced by, for example, a visual transformer [26] or another so-called predictive algorithm, for example, based on a recurrent network [42] and similar. Any reconstruction model that is able to transform the input image into its more ideal version without irregularities can be used.

We present the results of the proposed U2S-CNN in detail, from anomaly detection to a defect classification system. All results and visualizations to illustrate the reliability of U2S-CNN can be accessed in a GitHub repository. The first two phases are reaching industry-acceptable results. Not enough sufficient real results were obtained in the classification phase. The main issue is the problem of defect detection, where defects have very different patterns, locations, colors, etc. This is mainly the case of scratches, which are characterized as long and very thin patterns. Also, in this process, the input resolution 224 × 224 × 3 is used in the autoencoder. Higher resolutions can significantly improve this. The second issue is data quality, which is crucial in this type of task. In further research, attention has to be focused on the area of lighting conditions for scanning and inspecting an object and surfaces or parts [43]. The full evaluation of various lighting conditions was not in the scope of this work, and we will focus on it in future work. In the case of non-compliance with the conditions, these anomalies can be assimilated and may not be detected in the reconstruction process.

The purpose of this work is to demonstrate the proof of concept, focusing on defining the exact area of defects and specifying the defect area more precisely than just obtaining bounding boxes from standard detectors such as YOLO methods. From the point of view of the application of U-nets, transformers, and autoencoders, these methods provide only the basic truth areas. Also, the labels are not included in their results. Our system assigns labels and separates anomaly areas of specific defects. As a result, the proposed system combines the possibilities of two methods into one system and reaches the advantages of both approaches. Our assumption is a more feasible use of defect detection based on reconstruction in the first step due to the fundamental lack of strictly defined shapes of defects. These methods work, such as unsupervised approaches without labeled data. This way is not a problem with datasets with many different types of defects. 

## Figures and Tables

**Table 1:** Detection approaches with metrics.

Paper	Method	Metrics
[18]	Faster R-CNN	47.00–97.80% (Precision)
[20]	Autoencoder, classification	84.74–89.19% (Accuracy)
[17]	Improved Faster R-CNN	85.20% (Precision)
[16]	Fast R-CNN	72.90% (Precision)
[19]	Faster R-CNN	86.87–89.16% (Accuracy)
[15]	R-CNN	97–99% (Accuracy)
[21]	Segmentation (DeepLabv3+, U-net)	96.10–99.90% (Precision)
[22]	Mixed supervision	91.88–100.00% (Precision)
[23]	CS-ResNet	0.95 (Sensitivity, 𝛼 = 1)
[24]	U-net (Xception)	0.87 (Precision)
[25]	Convolutional autoencoder	0.9847 (Precision)
[26]	UTRAD (visual transformer)	78.9–100.0%

**Table 2:** Autoencoder summary.

Parameters	Number
Total params:	6,218,243
Trainable params:	6,212,355
Non-trainable params:	5888

**Table 3:** Training and validation dataset.

Category	No. of Training Samples	No. of Validation Samples
Damaged edge	124	25
Edge	123	28
Scratch	198	42
Surface damage	176	33
Teeth	193	42
Thread hole	167	35
Sum	981	205

**Table 4:** Xception model summary.

Parameters	Number
Total params:	20,873,774
Trainable params:	20,819,246
Non-trainable params:	54,528

**Table 5:** The evaluation of U2S-CNN.

The Type of Images	No. Samples	Detected Regions		RoI Labels		No. of Undetected Areas	No. of Damaged Areas
Correct samples (Training dataset)	78	All:	423	Good:	7	0	0
		RoIs:	16	Wrong:	9	0	0
Tested samples	61	All:	1920	Good:	108	142	205
		RoIs:	177	Wrong:	69		

## Data Availability

The datasets generated and/or analyzed during this study are not publicly available as they contain data about industrial objects (gearwheels) but are available from the corresponding author upon reasonable request.
